# Association of Lipoprotein‐Associated Phospholipase A2 and Lipoprotein(a) With the Risk of Recurrence Stroke in Patients With Acute Ischemic Stroke

**DOI:** 10.1002/jcla.25120

**Published:** 2024-12-03

**Authors:** Yu Feng, Shenyang Zhang, Hailiang Li, Hao Li, Ruiguo Dong, Shiguang Zhu, Yanlong Zhou

**Affiliations:** ^1^ Department of Neurology Affiliated Hospital of Xuzhou Medical University Xuzhou Jiangsu China; ^2^ Xuzhou Medical University Xuzhou Jiangsu China

**Keywords:** acute ischemic stroke, lipoprotein(a), lipoprotein‐associated phospholipase A2, major adverse cerebrovascular event, prognosis

## Abstract

**Objective:**

It is still a major global challenge to reduce the high morbidity and mortality of acute ischemic stroke (AIS) and improve the prognosis of patients. This study aims to investigate the prognostic value of lipoprotein‐associated phospholipase A2 (Lp‐PLA2) combined with lipoprotein(a) (Lp(a)) for long‐term stroke recurrence in patients with AIS.

**Methods:**

This study included 580 patients with AIS. Assessment of Lp‐PLA2 and Lp(a) levels was conducted upon patient admission. Continuous monitoring over the long term categorized stroke recurrence as an endpoint. Patients were categorized based on these identified thresholds to compare the risk of stroke recurrence: high Lp‐PLA2 and high Lp(a), high Lp‐PLA2 and low Lp(a), low Lp‐PLA2 and high Lp(a), and low Lp‐PLA2 combined with low Lp(a).

**Results:**

Among the 580 participants, 101 individuals (17.41%) experienced stroke recurrence within the 2‐year follow‐up. The majority were male (61.39%), with a median age of 62 years (interquartile range: 55–69.5). Factors independently associated with heightened the risk of recurrence stroke comprised age (hazard ratio [HR], 1.025; *p* = 0.021), diabetes mellitus (HR, 1.751; *p* = 0.007), Lp‐PLA2 (HR, 1.004; *p* < 0.001), and Lp(a) (HR, 1.002; *p* < 0.001). Noteworthy is that the combination of Lp‐PLA2 and Lp(a) displayed superior predictive efficacy for long‐term stroke recurrence risk in AIS patients compared to individual factors.

**Conclusion:**

This investigation underscores the potential advantage of leveraging the combined impact of Lp‐PLA2 in conjunction with Lp(a) as a more precise and cost‐effective predictive tool for the risk of recurrence stroke in patients with AIS.

## Graphical abstract

Lipoprotein‐associated phospholipase A2 and lipoprotein(a) can be used to predict major adverse cerebrovascular events in patients with acute ischemic stroke. The combined impact of Lp‐PLA2 in conjunction with Lp(a) as a more precise and cost‐effective predictive tool for the risk of recurrence stroke in patients with AIS. Lp‐PLA2 and Lp(a) are independently associated with major adverse cerebrovascular events in patients with acute ischemic stroke. Lp‐PLA2 and Lp(a) were simple and easy to obtain and can be considered as a routine test.

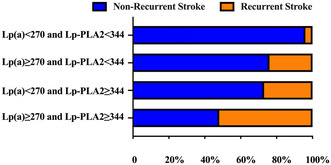

## 1 Introduction

Stroke is a leading cause of morbidity and mortality and a major cause of long‐term disability [1]. However, despite appropriate treatment, some patients with acute ischemic stroke (AIS) experience recurrent adverse cerebrovascular events, especially the recurrence of stroke has a great disability rate, which reduces the quality of life of patients and increases the global economic burden [2].

Previous research has confirmed that within atherosclerotic plaques, inflammatory cells express an enzyme known as lipoprotein‐associated phospholipase A2 (Lp‐PLA2) [1, 2, 3, 4], often referred to as platelet‐activating factor acetylhydrolase. Also present is lipoprotein(a) (Lp(a)), a lipoprotein formed through the union of apolipoprotein B‐100 (apo B‐100) with apolipoprotein(a), both exhibiting uncertain physiological functionalities [5]. On the other hand, Lp(a) and Lp‐PLA2 can participate in the occurrence of cardiovascular and cerebrovascular diseases by modulating oxidized phospholipids (OxPLs) [6, 7, 8]. While both Lp‐PLA2 and Lp(a) are acknowledged as fundamental risk factors for acute ischemic cerebrovascular disease [6, 7, 8, 9, 10, 11], few studies have conclusively associated them with the protracted risk of recurrent strokes in individuals affected by acute ischemic cerebrovascular disease [12, 13, 14]. Earlier research predominantly focused on individual evaluations of either Lp(a) or Lp‐PLA2 and their relationship with the potential for stroke recurrence among individuals facing acute cerebrovascular disease. Based on the above pathogenic effects of Lp‐PLA2 and Lp(a) on cardiovascular and cerebrovascular diseases, we hypothesis whether Lp‐PLA2 combined with Lp(a) is expected to be a better biomarker. Hence, this study primarily aims to explore whether concurrently assessing Lp‐PLA2 and Lp(a) yields additional predictive insights into predicting recurrent stroke risk following discharge in patients with AIS, compared to solely relying on Lp‐PLA2 or Lp(a) as distinct indicators, and ultimately improve the clinical prognosis of patients with AIS.

## 2 Materials and Methods

### 2.1 Population Characteristics

In this retrospective investigation, information concerning patients diagnosed with acute ischemic stroke (AIS) and admitted to the Affiliated Hospital of Xuzhou Medical University from August 2018 to August 2020 was subject to scrutiny. Upon arrival at the hospital's emergency department, all patients underwent treatment involving clopidogrel and aspirin [9]. The study rigorously complied with the ethical principles outlined in the Declaration of Helsinki and secured ethical endorsement from the Ethics Committee of the Affiliated Hospital of Xuzhou Medical University. Given the retrospective design of the paper, the necessity for obtaining specific informed consent was omitted.

The diagnosis of AIS was established based on the persistence or rapid progression of neurological symptoms, accompanied by corresponding ischemic changes detected via magnetic resonance imaging (MRI) or computed tomography (CT). Validating stroke classifications was accomplished through magnetic resonance angiography (MRA) or CT angiography (CTA), in addition to electrocardiography and echocardiography. Classification of AIS cases aligned with the initial TOAST criteria [15], encompassing five main classifications: large artery atherosclerosis (LAA), cardioembolism (CE), small artery occlusion (SAO), stroke of other determined cause (SOD), and stroke of undetermined cause (SUD), which cover various origins, unidentified sources, or incomplete evaluations.

Exclusion criteria for AIS patients included: (1) administering clopidogrel and aspirin after 4.5 h from symptom onset; (2) lack of clinical data; (3) Attrition within 2 years after leaving the hospital; (4) intracranial hemorrhage and intracranial space‐occupying lesions confirmed by CT or MRI; (5) transient ischemic attack (TIA); (6) intravenous thrombolysis or intravascular therapy; (7) complications of infection, malignant tumor, severe cardiac insufficiency, liver and kidney diseases, and autoimmune diseases; and (8) death during hospitalization.

### 2.2 Data Collection

The basic information of patients (age, sex, and contact information) and risk factors for cerebrovascular disease (hypertension, diabetes, heart disease, time from symptom onset to antiplatelet therapy, and prior medications) were collected. The definition of risk factors is as follows: The diagnostic criteria used for hypertension adhered to the 2010 Chinese guidelines for managing hypertension, while the determination of diabetes conformed to the guidelines set forth in 2013 for the prevention and control of type 2 diabetes in China. Heart disease encompassed any documented history or clinical demonstration of conditions like myocardial infarction, angina pectoris, congestive heart failure, or arrhythmia. Upon admission, all patients at our medical center underwent comprehensive assessments, including routine blood analysis, biochemical evaluations, coagulation function assessments, and the measurement of Lp‐PLA2 and Lp(a) levels via immunoturbidimetry on venous blood samples.

The National Institute of Health Stroke Scale (NIHSS) score was utilized to assess the extent of neurologic impairment [16].

### 2.3 Follow‐Up

Patients received follow‐up care via telephone or outpatient services within the initial month post‐discharge, followed by subsequent assessments every 3 months. They or their caregivers were asked about any stroke occurrences or new neurologic symptoms. Confirmation of recurrent AIS cases relied on relevant clinical symptoms or neuroimaging data (CT or MRI) assessed by neurologists. Additionally, patients continued their prescribed AIS prevention medications specific to their stroke subtype during the follow‐up period. Based on long‐term follow‐up outcomes, patients were categorized into either the major adverse cerebrovascular event (MACE) group or the non‐MACE group.

### 2.4 Statistical Procedures

The Kolmogorov–Smirnov test was employed to assess data normality. Non‐normally distributed continuous variables were presented as the median with interquartile range (IQR). Skewed continuous variables underwent evaluation via the Mann–Whitney *U* test, while categorical variables were analyzed using the chi‐squared test or Fisher's exact test. For the identification of stroke recurrence risk factors in AIS patients, univariate Cox regression analysis was conducted, incorporating variables that exhibited a significance level of *p* < 0.05 in the multivariate Cox regression analysis model. The forward likelihood ratio method was used, considering potential collinearity among neutrophils, lymphocytes, platelets, neutrophil‐to‐lymphocyte ratio (NLR), and platelet‐to‐lymphocyte ratio (PLR). The assessment of Lp‐PLA2 and Lp(a) values' predictability for AIS patient outcomes involved utilizing the receiver operating characteristic (ROC) curve. Binary logistic regression analysis was used to calculate the predictive probability of Lp‐PLA2 combined with LP (a) in the diagnosis of MACE, Logit (*P*) = −3.963 + 0.005*Lp‐PLA2 + 0.003*LP (a). To compare the predictiveness, the De–Long test was implemented. For the comparison of survival rates among non‐MACE groups, the Kaplan–Meier method was applied. Evaluating associations between variables employed Spearman correlation analysis. The statistical significance was established at a two‐sided *p* ≤ 0.05. The data analysis was performed using Statistica 23.0 (StatSoft Inc., Tulsa, OK, USA), and graphical representations were generated using GraphPad Prism 9.0 (GraphPad Software, San Diego, CA, USA).

## 3 Results

### 3.1 Baseline Characteristics of Patients With ICVD

Table 1 presents a comprehensive summary of the initial clinical attributes observed in the patient cohort. The research encompassed 580 participants, with a median age of 64 years (interquartile range: 55–71.25), with 66.90% being male. Median Lp‐PLA2 levels were 134 (82–280) ng/mL, and median Lp(a) levels were 260 (126–383.5) mg/L in the sample. Detailed baseline clinical characteristics, including lipid measurements, homocysteine (HCY) levels, and more, are delineated in Table 1. Over the 2‐year follow‐up, 101 individuals experienced recurrent strokes.

**TABLE 1:** Basic Clinical Characteristics Between major adverse cardiovascular events (MACE) Groups and Non‐MACEs Groups in Patients with acute ischemic stroke (AIS).

Basic clinical characteristics	MACEs group, *n* = 101	Non‐MACEs group, *n* = 479	*p*
Demographics			
Age, years	67 (60,72)	63 (55,71)	0.003
Male gender, *n* (%)	62 (61.39)	336 (70.15)	0.085
Smoking, *n* (%)	29 (28.72)	91 (19.00)	0.028
Hypertension, *n* (%)	66 (65.35)	287 (59.92)	0.310
Diabetes mellitus, *n* (%)	36 (35.64)	108 (22.55)	0.006
Atrial fibrillation, *n* (%)	16 (15.84)	43 (8.98)	0.038
Coronary artery disease, *n* (%)	21 (20.79)	57 (11.90)	0.017
Time from symptom onset to antiplatelet therapy (*h*)	3.0 (2.0, 4.0)	2.5 (2.0, 3.5)	0.001
Prior medications			
Anticoagulation, *n* (%)	13 (12.87)	40 (8.35)	0.152
Antiplatelet, *n* (%)	14 (13.86)	31 (6.47)	0.012
Statin therapy, *n* (%)	19 (18.81)	52 (10.86)	0.027
Laboratory parameters			
Hemoglobin (g/L)	141 (129, 152)	141 (131, 150)	0.742
Total cholesterol (mmol/L)	4.79 (3.85, 5.71)	4.39 (3.77, 5.01)	0.004
Triglyceride (mmol/L)	1.43 (1.05, 1.86)	1.27 (0.94, 1.77)	0.033
HDL cholesterol (mmol/L)	1.09 (0.95, 1.30)	1.13 (0.95, 1.36)	0.695
LDL cholesterol (mmol/L)	3.04 (2.15, 3.77)	2.59 (2.08, 3.26)	0.001
White blood cells, 10^3^/μL	6.40 (5.30, 7.90)	6.20 (5.20, 7.40)	0.367
Neutrophils, 10^3^/μL	4.14 (3.43, 5.38)	4.07 (3.18, 5.07)	0.188
Lymphocytes, 10^3^/μL	1.50 (1.20, 1.80)	1.60 (1.20, 1.90)	0.434
Platelets, 10^3^/μL	222 (196, 250)	216 (192, 243)	0.110
C‐reactive protein (mg/L)	8.92 (4.52,10.71)	7.76 (4.18, 9.37)	0.037
NLR	2.82 (2.15, 3.80)	2.54 (1.84, 3.43)	0.049
PLR	149 (115, 183)	139 (111, 178)	0.117
Homocysteine (μmol/L)	13.90 (10.65, 17.00)	13.34 (10.65, 17.06)	0.001
Lp‐PLA2 (ng/ml)	368 (180, 460)	173 (106, 288)	< 0.001
Lp(a) (mg/L)	312 (211, 518)	195 (130, 277)	< 0.001
TOAST, *n* (%)			
Large artery atherosclerosis, *n* (%)	58 (57.43)	244 (50.94)	0.236
Small artery occlusion, *n* (%)	33 (32.67)	206 (43.01)	0.055
Cardioembolism, *n* (%)	5 (4.95)	17 (3.55)	0.503
Other, *n* (%)	4 (3.96)	12 (2.51)	0.417
Undetermined, *n* (%)	1 (0.99)	0 (0)	0.174
Admission NIHSS score	5 (3, 7)	4 (2, 6)	< 0.001

*Note:* Definitions were outlined for Lp‐PLA2 (lipoprotein‐associated phospholipase A2), Lp(a) (lipoprotein(a)), HDL cholesterol (high‐density lipoprotein cholesterol), LDL cholesterol (low‐density lipoprotein cholesterol), NLR (neutrophil‐to‐lymphocyte ratio), PLR (platelet‐to‐lymphocyte ratio), LAA (large artery atherosclerosis), CE (cardioembolism), SAO (small artery occlusion), SOD (stroke of other determined cause), and SUD (stroke of undetermined cause). The statistical analyses encompassed the utilization of the Mann–Whitney *U* test and chi‐square test.

### 3.2 Correlation With the Recurrence of Ischemic Cerebrovascular Disease

The data in Table 2 outlines hazard ratios (HR) pertaining to established risk factors associated with recurring strokes. Following adjustment of these variables via multivariate analysis using Cox proportional hazards regression model, age (HR, 1.031; 95% CI, 1.010–1.053; *p* = 0.031), diabetes mellitus (HR, 1.790; 95% CI, 1.189–2.694; *p* = 0.005), Lp‐PLA2 ≥ 134 (median) (HR, 1.824; 95% CI, 1.067–3.121; *p* = 0.028), and Lp(a) ≥ 260 (median) (HR, 2.294; 95% CI, 1.932–3.646; *p* = 0.041) emerged significantly as risk factors for recurring strokes.

**TABLE 2:** Both univariate and multivariable Cox proportional hazard regression analyses were conducted to establish the incidence of MACE within a 2‐year period post‐hospital discharge among individuals diagnosed with acute ischemic stroke (AIS).

Long‐term recurrent stroke								
	Univariate analysis				Multivariable analysis			
	*p*	HR	95% CI		*p*	HR	95% CI	
Age, years	0.003	1.031	1.011	1.052	0.031	1.031	1.010	1.053
Smoking, *n* (%)	0.027	1.626	1.057	2.502				
Diabetes mellitus, *n* (%)	0.006	1.761	1.172	2.646	0.005	1.790	1.189	2.694
Atrial fibrillation, *n* (%)	0.028	1.821	1.067	3.107				
Coronary artery disease, *n* (%)	0.016	1.802	1.114	2.914				
Time from symptom onset to antiplatelet therapy (hours)	0.001	1.337	1.120	1.596				
Antiplatelet, *n* (%)	0.012	1.756	1.173	3.627				
Statin therapy, *n* (%)	0.027	1.066	2.893	1.756				
Total cholesterol (mmol/L)	0.820	0.998	0.981	1.015				
Triglyceride (mmol/L)	0.467	1.074	0.886	1.301				
LDL cholesterol (mmol/L)	< 0.001	1.426	1.169	1.739				
C‐reactive protein (mg/L)	0.197	1.022	0.987	1.108				
NLR	0.285	1.046	0.963	1.136				
Homocysteine (μmol/L)	0.202	1.017	0.991	1.044				
Lp‐PLA2 ≥ 134 (median) (ng/mL)	0.007	2.074	1.216	3.539	0.028	1.824	1.067	3.121
Lp(a) ≥ 260 (median) (mg/L)	0.016	2.983	2.017	4.557	0.041	2.294	1.932	3.646
Admission NIHSS score	< 0.001	1.079	1.035	1.125				

Abbreviations: LDL cholesterol, low‐density lipoprotein cholesterol; Lp(a), lipoprotein(a); Lp‐PLA2, Lipoprotein‐associated phospholipase A2; NLR, neutrophil‐to‐lymphocyte ratio.

### 3.3 The Study Explored the Potential of Lp(a), Lp‐PLA2, or Their Combination in Predicting Stroke Recurrence Among AIS Patients

**FIGURE 1:**
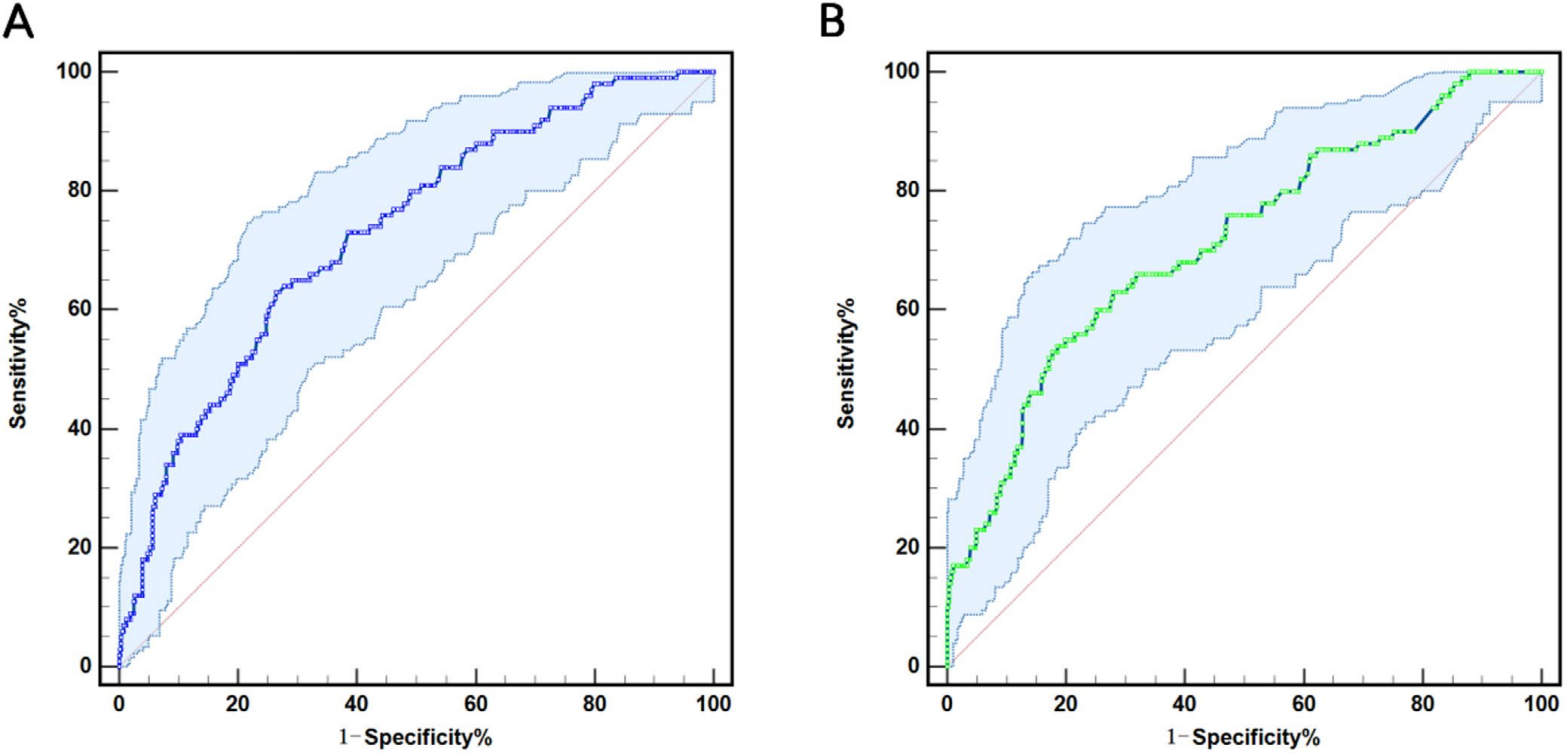
Charts illustrating ROC curves (A) ROC curve for Lp(a) to predict stroke recurrence; (B) ROC curve for Lp‐PLA2 to predict stroke recurrence.

Figure 1 and Table 3 demonstrate the predictive prowess of Lp(a) and Lp‐PLA2 in foreseeing stroke recurrence among AIS patients using ROC curves. The findings emphasized the substantial predictive efficacy of both Lp(a) (AUC 0.730 [95% CI 0.678–0.783]; *p* < 0.001) and Lp‐PLA2 (AUC 0.716 [95% CI 0.659–0.773]; *p* < 0.001) for anticipating stroke recurrence in AIS patients. At an Lp(a) cutoff of 270 mg/L, the sensitivity was 63.0%, and specificity was 73.5% in predicting long‐term MACEs in AIS patients. At the Lp‐PLA2 cutoff value of 334 ng/mL, the sensitivity was 54.0%, and the specificity was 81.4% in predicting long‐term MACEs in patients with AIS.

**TABLE 3:** Receiver operating characteristic curve analysis of lipoprotein‐associated phospholipase A2 or lipoprotein(a) and their combination in predicting MACE in patients with AIS after discharge.

	Cutoff	AUC	95% CI	*p*
Lp(a)	270	0.730	0.678–0.783	< 0.001
Lp‐PLA2	334	0.716	0.659–0.773	< 0.001
Lp‐PLA2‐Lp(a)	0.091	0.794	0.753–0.836	< 0.001

Abbreviations: Lp(a), Lipoprotein(a); Lp‐PLA2, Lipoprotein‐associated phospholipase A2.

**FIGURE 2:**
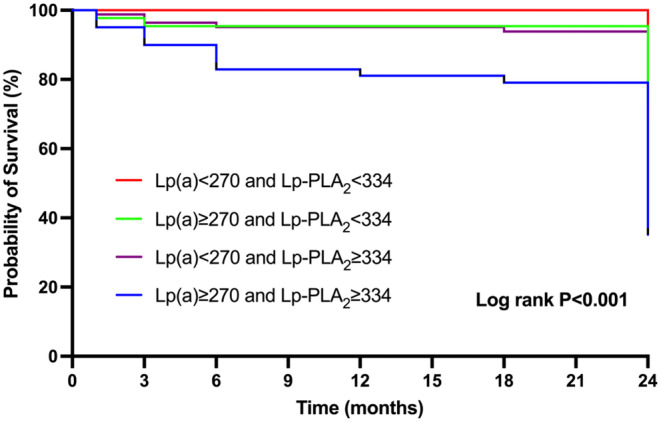
Kaplan–Meier curve analysis assessed the cumulative risks across Lp(a) and Lp‐PLA2 categories.

Patients were grouped into four subcategories based on LP(a) (cutoff value, 270 mg/L) and Lp‐PLA2 (cutoff value, 334 ng/mL): Group 1 (Lp(a) < 270 mg/L and Lp‐PLA2 < 334 ng/mL), Group 2 (Lp(a) ≥ 270 mg/L and Lp‐PLA2 < 334 ng/mL), Group 3 (Lp(a) < 270 mg/L and Lp‐PLA2 ≥ 334 ng/mL), and Group 4 (Lp(a) ≥ 270 mg/L and Lp‐PLA2 ≥ 334 ng/mL). The recurrence rate of stroke in each group is shown in Table 4. Evaluations were carried out within these four categories to appraise the likelihood of stroke recurrence (refer to Table 5). In comparison with Group 1 (Lp(a) < 270 mg/L and Lp‐PLA2 < 334 ng/mL), the risk of stroke recurrence was 5.766 times higher in Group 2 (Lp(a) ≥ 270 mg/L and Lp‐PLA2 < 334 ng/mL), 6.547 times higher in Group 3 (Lp(a) < 270 mg/L and Lp‐PLA2 ≥ 334 ng/mL), and 14.894 times higher in Group 4 (Lp(a) ≥ 270 mg/L and Lp‐PLA2 ≥ 334 ng/mL), all statistically significant (*p* < 0.001). Analysis using Kaplan–Meier survival curves revealed a significantly elevated incidence of extended‐term stroke recurrence among patients in Group 4 (log‐rank *p* < 0.001) (visualized in Figure 2).

**TABLE 4:** Clinical outcomes at 2‐year follow‐up after discharge in patients with AIS.

	Total	Recurrent stroke at 2 year *n* (%)
Lp(a) < 270 and Lp‐PLA2 < 334	303	14 (4)
Lp(a) ≥ 270 and Lp‐PLA2 < 334	132	32 (24)
Lp(a) < 270 and Lp‐PLA2 ≥ 334	84	23 (27)
Lp(a) ≥ 270 and Lp‐PLA2 ≥ 334	61	32 (52)

Abbreviations: Lp(a), Lipoprotein(a); Lp‐PLA2, Lipoprotein‐associated phospholipase A2.

**TABLE 5:** Comparison of long‐term MACE risk in different subgroups of patients after discharge.

	*β*	Wald*χ* ^2^	HR	95% CI	*p*
Lp(a) ≥ 270 and Lp‐PLA2 ≥ 334 vs. Lp(a) < 270 and Lp‐PLA2 < 334	2.707	70.990	14.984	7.983–28.126	< 0.001
Lp(a) ≥ 270 and Lp‐PLA2 < 334 vs. Lp(a) < 270 and Lp‐PLA2 < 334	1.752	29.879	5.766	3.077–10.808	< 0.001
Lp(a) < 270 and Lp‐PLA2 ≥ 334 vs. Lp(a) < 270 and Lp‐PLA2 < 334	1.879	30.709	6.547	3.368–12.725	< 0.001

Abbreviations: Lp(a), Lipoprotein(a); *p*‐PLA2, Lipoprotein‐associated phospholipase A2.

**FIGURE 3:**
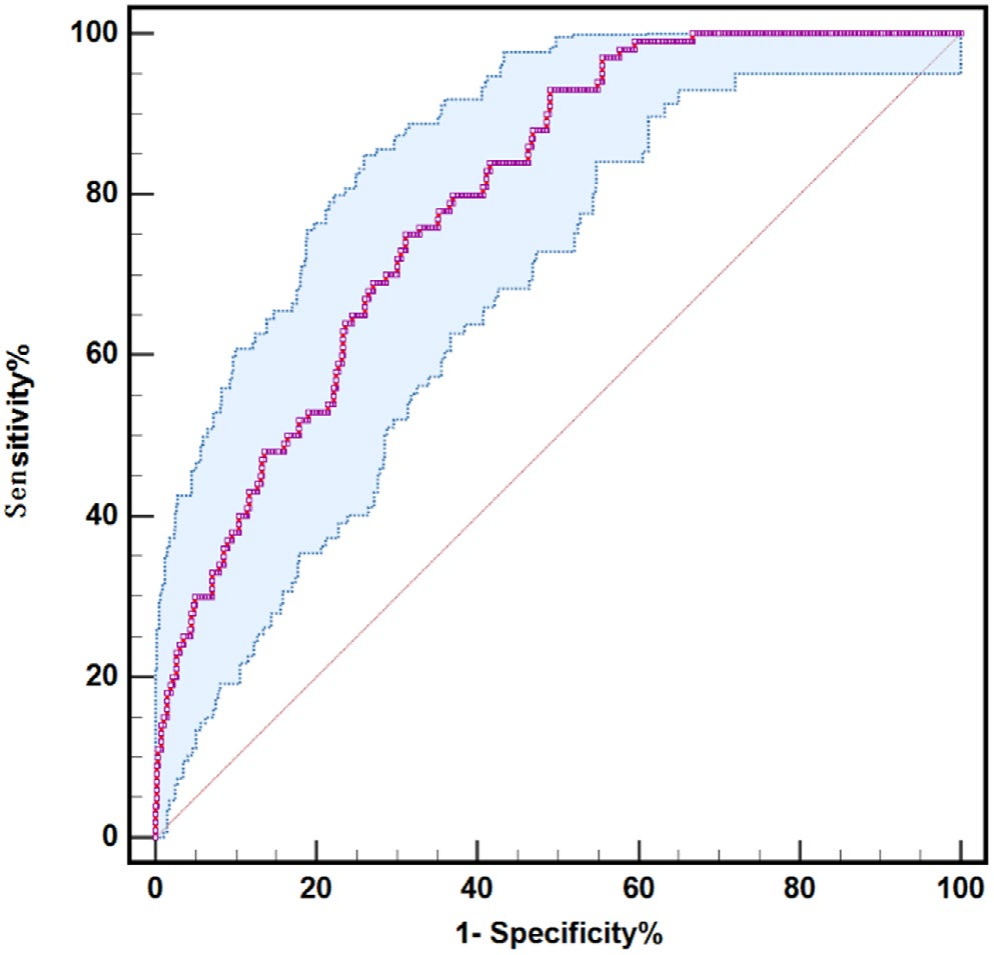
The aim was to evaluate the collective predictive capacity of Lp(a) and Lp‐PLA2 through receiver operating characteristic (ROC) curve analysis. This evaluation aimed to gauge the long‐term risk of stroke recurrence in individuals diagnosed with acute ischemic stroke (AIS).

Using recurring strokes as the clinical observation endpoint, the ROC curve assessed the predictive value of combining Lp(a) with Lp‐PLA2 for stroke recurrence risk in AIS patients. At the combined Lp(a) and Lp‐PLA2 value of 0.091, the exhibited sensitivity was 93.0%, specificity was 50.9% in predicting long‐term MACEs in AIS patients, and the AUC was 0.794 [95% CI 0.753–0.836] *p* < 0.001 (Figure 3 and Table 3). The De–Long test indicated that Lp‐PLA2 combined with Lp(a) had better predictive efficacy for the long‐term poor prognosis of patients with AIS, which was better than Lp‐PLA2 (AUC 0.794 [95% CI 0.753–0.836] vs. AUC 0.716 [95% CI 0.659–0.773]; *p* = 0.001) or Lp(a) (AUC 0.794 [95% CI 0.753–0.836] vs. AUC 0.730 [95% CI 0.678–0.783]; *p* = 0.015) (Table 6).

**TABLE 6:** Receiver operating characteristic curve to evaluate lipoprotein‐associated phospholipase A2 or lipoprotein(a) and their combination have different predictive effects on stroke recurrence in patients with AIS.

	Area of difference	95% CI	*p*
Lp(a) vs. Lp‐PLA2	0.014	−0.068–0.100	0.711
Lp(a) vs. Lp‐PLA2‐Lp(a)	0.064	0.012–0.114	0.015
Lp‐PLA2 vs. Lp‐PLA2‐Lp(a)	0.078	0.039–0.119	0.001

Abbreviations: Lp(a), Lipoprotein(a); *p*‐PLA2, Lipoprotein‐associated phospholipase A2.

### 3.4 Association Among Lp(a), Lp‐PLA2, and NIHSS Score

The NIHSS score is currently one of the most commonly used risk stratification methods for AIS patients worldwide. Employing the NIHSS scale for evaluating severity and prognosis in patients with acute ischemic stroke (AIS), we conducted bivariate Spearman rank correlation analysis to investigate the interplay between Lp(a), Lp‐PLA2, and NIHSS scores. Our analysis revealed noteworthy positive associations between Lp(a) and Lp‐PLA2 with the NIHSS scores, demonstrating correlation coefficients of 0.094 (*p* = 0.024) and 0.207 (*p* < 0.001), respectively. The correlation coefficient between Lp(a) and NIHSS is very low.

## 4 Discussion

Prolonged inflammation has been linked to an augmented burden of atherosclerotic disease and an increased susceptibility to ischemic stroke [17]. Both Lp(a) and Lp‐PLA2 have been implicated in the inflammatory response following an ischemic stroke [14]. Lp‐PLA2, an enzyme derived from leukocytes, is released in response to inflammation and is thought to actively contribute to atherogenesis by breaking down low‐density lipoprotein (LDL) into oxidized phospholipids. These phospholipids exhibit direct inflammatory traits, triggering downstream proinflammatory signaling effects. The prevailing agreement suggests that Lp‐PLA2 catalyzes the breakdown of oxidized phospholipids, creating proinflammatory compounds that contribute to endothelial dysfunction, inflammation within plaques, and the development of necrotic cores within them. This process establishes a connection between the oxidative modification of LDL and the inflammatory reactions within the arterial intima [18]. Lp(a), characterized as a genetic variant of LDL, transports apolipoprotein (a) linked to Apo B‐100 via a disulfide bond, displaying structural resemblances to plasminogen. This structural analogy endows it with atherogenic and atherothrombotic properties [19]. The precise mechanisms by which Lp(a) mediates atherogenicity remain uncertain. Existing data indicates that LP(a) functions as a risk factor for atherosclerotic cardiovascular disease (ASCVD), encompassing myocardial infarction and ischemic stroke [20, 21, 22]. Inflammatory markers have been linked to recurrent vascular events [23]. However, the link between LP(a), Lp‐PLA2, and the recurrence of cerebrovascular disease has been scarcely investigated. An analysis involving 79,036 participants from 32 distinct prospective studies consistently revealed that both Lp‐PLA2 activity and mass were consistently associated with an increased risk of stroke and myocardial infarction [18]. Wang's study established a relationship between Lp‐PLA2‐A levels measured in the acute phase after TIA or minor stroke and the immediate risk of recurrent vascular events [14]. Furthermore, Zheng's research underscored that heightened serum levels of Lp(a) upon admission were associated with short‐term stroke recurrence [24].

In our investigation, we confirmed that age (HR, 1.031; 95% CI, 1.010–1.053; *p* = 0.031), diabetes mellitus (HR, 1.790; 95% CI, 1.189–2.694; *p* = 0.005), Lp‐PLA2 ≥ 134 (median) (HR, 1.824; 95% CI, 1.067–3.121; *p* = 0.028), and Lp(a) ≥ 260 (median) (HR, 2.294; 95% CI, 1.932–3.646; *p* = 0.041) were independently associated with an increased risk of stroke recurrence in AIS patients. These results align with previous study findings [25, 26, 27]. Both Lp(a) and Lp‐PLA2 demonstrated significant predictive efficacy for the enduring probability of recurrent stroke among AIS patients.

Subgroup analysis highlighted significant differences in the absolute risks of recurrent cerebrovascular events among the four groups, based on elevations in either LP(a) or Lp‐PLA2. When Lp(a) < 270 mg/L and Lp‐PLA2 < 334 ng/mL, we found the lowest risk of recurrent cerebrovascular events in 2 years. When only one of them was high, we found a moderate risk of recurrence cerebrovascular events. Surprisingly, over a span of 2 years, individuals with both high Lp(a) (≥ 270 mg/L) and elevated Lp‐PLA2 (≥ 334 ng/mL) experienced the highest rate of recurring cerebrovascular events. Our results confirm that heightened Lp‐PLA2 notably increases the risk of recurrent ischemic cerebrovascular disease, particularly when coupled with elevated levels of Lp(a). These findings emphasize the crucial necessity for enhanced monitoring of Lp‐PLA2 levels, especially in patients displaying elevated LP(a), acknowledged as an intrinsic, unchangeable, and primarily genetically predetermined risk factor [13]. The particular function of Lp(a)‐related Lp‐PLA2 remains insufficiently explored. Among individuals displaying heightened plasma Lp(a) concentrations, Lp(a)‐Lp‐PLA2 may function similarly to LDL‐Lp‐PLA2 within the arterial wall [4, 28, 29]. Lp(a) tends to accumulate more within lesions in proportion to LDL, demonstrating a strong affinity for lesion components that corresponds to its plasma levels [30]. Some literature suggests the potential pivotal role of oxidized phospholipid (OxPL) content within Lp(a) in its functionality [5]. Within the intima, Lp(a) might transport preexisting OxPLs, potentially undergoing further oxidative modifications, thereby enriching OxPLs [15]. Lp(a)‐bound OxPLs could then undergo hydrolysis by Lp(a)‐Lp‐PLA2, resulting in the generation of OxFFA and lyso‐PC [31], thereby significantly contributing to plaque formation. Therefore, through this mechanism, Lp(a)‐Lp‐PLA2 could profoundly impact the biological effects of oxidized Lp(a) within the arterial wall, fostering atherogenesis [32, 33]. These mechanisms could explain the higher recurrence rate of stroke observed in patients with Lp(a) ≥ 270 mg/L and Lp‐PLA2 ≥ 334 ng/mL.

The ROC curve confirmed the superior predictive capability of combining Lp(a) and Lp‐PLA2 in assessing the risk of stroke recurrence among AIS patients compared to their individual predictive capacities. The correlation analysis indicated a direct link between Lp(a), Lp‐PLA2, and the NIHSS score. This relationship might partly justify the utilization of Lp‐PLA2, Lp(a), and their combined values as prognostic factors for an extended period probability of recurrent stroke in patients with AIS.

The study encountered several limitations. Initially, our focus was exclusively on predicting long‐term stroke recurrence risk in AIS patients based on the concentrations of Lp‐PLA2 and Lp(a) upon emergency admission. Multiple blood draws to track Lp(a) and Lp‐PLA2 throughout the disease progression were not performed. We aimed to investigate whether diverse trends in Lp(a) and Lp‐PLA2 could enhance estimation of the risk of stroke recurrence. Secondly, measurements of both Lp‐PLA2 and Lp(a) were conducted post‐stroke, potentially failing to accurately represent pre‐stroke levels.

## 5 Conclusions

Both Lp(a) and Lp‐PLA2 were identified as autonomous predictors of extended‐term MACE (major adverse cardiovascular events) in AIS patients. Individuals with elevated levels of Lp(a) and Lp‐PLA2 upon emergency admission, particularly those with increased Lp(a) combined with heightened Lp‐PLA2, faced significantly increased risks of long‐term MACE. The predictive sensitivity and specificity of Lp(a) and Lp‐PLA2 separately in forecasting long‐term MACE among AIS patients were relatively lower. The predictive value of Lp(a) combined with Lp‐PLA2 for MACE in the target population was significantly improved, suggesting that Lp(a) combined with Lp‐PLA2 can serve as a superior indicator for assessing long‐term stroke recurrence risk in AIS patients.

## Author Contributions

All authors made a considerable contribution to the work reported, whether that is in the conception, study design, execution, acquisition of data, analysis, and interpretation, or all these areas; took part in drafting, revising, or critically reviewing the article; gave final approval of the version to be submitted for publication; have agreed on the journal to which the article has been submitted; and agreed to be accountable for all aspects of the work.

## Ethics Statement

This study was conducted in accordance with the Declaration of Helsinki and was approved by the Local Ethics Committee of the Affiliated Hospital of Xuzhou Medical University (ethics approval number XYFY2019‐KL042‐01). Considering the retrospective nature of the study, the requirement for patient consent was waived by the ethics committee. All patient data were anonymized or maintained confidentially.

## Conflicts of Interest

The authors declare no conflicts of interest.

## Data Availability

The data that support the findings of this study are available on request from the corresponding author. The data are not publicly available due to privacy or ethical restrictions.
